# Developing a provincial patient support network for children and families affected by Tourette syndrome and/or obsessive–compulsive disorder: results of a stakeholder consultation

**DOI:** 10.1186/s13034-021-00383-5

**Published:** 2021-06-16

**Authors:** Julian Fletcher, Gina Dimitropoulos, Davide Martino, Gabrielle Wilcox, Frank MacMaster, Paul Arnold, Tamara Pringsheim

**Affiliations:** 1 Department of Clinical Neurosciences, Cumming School of Medicine, University of Calgary, Calgary, Canada; 2 Faculty of Social Work, University of Calgary, Calgary, Canada; 3 Werklund School of Education, University of Calgary, Calgary, Canada; 4 Department of Psychiatry, Cumming School of Medicine, University of Calgary, Calgary, Canada; 5 Mathison Centre for Mental Health Education and Research, 3280 Hospital Drive NW, Calgary, AB T2N 4Z6 Canada

**Keywords:** Obsessive–compulsive disorder, Tourette syndrome, Patient engagement, Health services planning

## Abstract

**Background:**

Tourette syndrome and OCD are disorders that frequently occur in children and cause a high level of disability. In Alberta there is a huge delivery gap in providing healthcare services for children with TS and OCD. A stakeholder consultation was performed to ascertain how service delivery could be improved across the province and to inform the development of a provincial information and support organization, the Tourette OCD Alberta Network.

**Methods:**

A mixed-methods study was employed: 10 parents were recruited for interview and 140 parents responded to a survey.

**Results:**

Qualitative data showed there was often an absence of a clear pathway to access healthcare for people with TS and OCD. The negative impact of not receiving treatment, information, and resources in a timely and prompt manner was also revealed. Good clinical practice existed across the province but too often it was hindered by a shortage of knowledge about TS and OCD. In schools, learning for students with TS and OCD was also impaired by educators’ lack of knowledge and preparedness in relation to the disorders.

**Conclusions:**

This study identified ways that challenges with healthcare access, school learning, and seeking information can be overcome. Skills-based training webinars, educational outreach in schools, and peer support were recognized as actions for improving healthcare outcomes for people with TS and OCD. The aim of the Tourette OCD Alberta Network is to provide services and support that directly address the healthcare service delivery shortfalls shown in this study.

**Supplementary Information:**

The online version contains supplementary material available at 10.1186/s13034-021-00383-5.

## Background

Tourette syndrome (TS) and obsessive compulsive disorder (OCD) are two common childhood onset disorders which frequently co-occur, with up to 50% of children with TS meeting diagnostic criteria for OCD [1], and approximately 20% of children with OCD experiencing tics [2]. Children and families affected by TS and OCD face two problems regarding treatment. First, access to specialized treatment is limited. These disorders cause a high level of disability and require specialized knowledge for diagnosis and treatment. Second, both TS and OCD have marked complexity. Children with TS and OCD experience disabling symptoms and have a high degree of neuropsychiatric and neurodevelopmental comorbidity, adding to the overall disease burden. The lifetime prevalence of any psychiatric comorbidity in people with TS is 85.7% [1]. In people with OCD, the prevalence of psychiatric comorbidity is also very high, with 62% having at least one comorbid psychiatric condition [3]. The medical and psychiatric complexity of children with TS and OCD makes providing effective and accessible comprehensive services challenging. Children with TS and OCD often need more than a physician, with other allied health professionals providing essential behavioural therapies and family support.

The province of Alberta in Canada consists of 4.4 million people spread over 661,848 km^2^. Health services are administered by a single provincial publicly funded entity, Alberta Health Services, which is divided into five zones: North, Edmonton, Central, Calgary and South. Children with TS and/or OCD in Alberta are subject to a tremendous service delivery gap, as the only specialized clinics for children with TS and OCD in Alberta are in the Calgary zone at the Alberta Children’s Hospital. These clinics do not currently have the infrastructure to support the province-wide multidisciplinary care needs of this population. In order to address this disparity, we received government funding to create the Tourette OCD Alberta Network. The main purpose of the Tourette OCD Alberta Network is to relieve the burden on children and families by increasing capacity and improving access to comprehensive patient- and family-centred care for children and youth with TS and OCD province wide.

We performed a stakeholder consultation with people with lived experience with TS and OCD in Alberta, to determine their service needs and inform the development of our organization. We believe that the information from this stakeholder consultation will be of interest to mental health researchers and others seeking to improve service delivery to individuals with TS and/or OCD or other mental health disorders in other locations.

## Methods

This study was approved by the University of Calgary Conjoint Health Research Ethics Board. This was a concurrent mixed-methods study, consisting of a survey and one-on-one interviews, with parents of children with TS and/or OCD from across Alberta. The survey and interviews were conducted simultaneously.

### Survey

Survey questions were developed by our multidisciplinary research team from the fields of neurology, psychiatry, psychology, nursing, social work, and education, and included individuals with lived experience with TS and OCD. Survey questions included multiple-choice questions and free text entry fields. Survey questions related to service utilization and perceived service and support needs (see Additional file 1 for full survey). Electronic consent was obtained from all survey participants prior to proceeding with the survey questions.

The survey data were collected from August 1 to October 31 of 2019. To disseminate the survey, we used several strategies. The survey was disseminated using a survey link by email to all members on the mailing list of the Alberta OCD Foundation, the Tourette Canada Edmonton chapter, and the Tourette Canada Calgary chapter, and links to the survey were also posted on their Facebook pages. Additionally, parents attending clinic appointments at the Alberta Children’s Hospital TS and OCD clinics were approached by the research coordinator and given a tablet to complete the survey.

### Interviews

The interview guide was developed by the research team. Ten parents were recruited for the interview by our research coordinator through existing patients at the Alberta Children’s Hospital TS and OCD clinics, with the goal of recruiting participants who resided in different parts of Alberta. To be eligible for inclusion, parents had to have a child formally diagnosed by a physician with TS and/or OCD and be able to participate in an interview in English. Interviews were conducted by telephone by our research coordinator, audiotaped and transcribed verbatim. All participants provided signed consent for their participation.

### Analysis

For the survey data, we evaluated the frequency of response choices for each question.

The interview transcripts were analyzed using thematic analysis. The analysis proceeded according to the steps outlined by Braun and Clarke [4]. First, the research team familiarized themselves with the data, by reading and re-reading the data and making notes of initial ideas. Initial codes were generated in a systematic fashion across the entire data set, with collation of data relevant to each code. The team then searched for themes and collated codes into potential themes. Themes were reviewed in relation to the coded extracts and the entire data set. The themes were finally defined and named. Two members of the research team each independently reviewed the data and performed the initial coding. The research team then met to discuss the coding, with any discrepancies discussed until consensus was reached. Consensus was always achieved through discussion of the transcripts. The team worked together to search for themes and to define and name these. NVivo11 software was used for the organization of the data.

## Results

### Survey

The survey was completed by 140 parents, with a mean age of respondents of 43 years. Geographically, responses were obtained from all five Alberta Health Services zones.

The different types of physicians providing on-going care (writing prescriptions and regular follow-up visits) are presented in Table 1. Over half (52%) of parents had taken their child to see an allied health care professional. Of these, the most commonly seen were psychologists (29%), mental health counsellors (20%), occupational therapists (17%), social workers (13%), and family therapists (11%). Other professionals frequently mentioned in the free text fields included naturopathic doctors, chiropractors, and massage therapists. How parents found out about allied health care providers is presented in Table 2.

**Table 1:** Type of physician providing on-going care for TS and/or OCD

Type of Physician	%
Psychiatrist	36
Pediatrician	20
Family Physician	16
Neurologist	11
Other specialist	7
No Physician	11

**Table 2:** Methods used to find additional health-care providers for TS and/or OCD

How healthcare providers were found	%
Physician referral	32
Internet search	19
Access Mental Health^a^	15
Friend recommendations	12
Patient organizations	11

^a^Access Mental Health is a self-referral program for provincially funded mental health service needs in the Calgary zone

Over half (56%) of parents took their children to fee-for-service allied health care professionals. The most commonly sought services from allied health care professionals by parents were family therapy (21%), parent training for aggressive behaviour (20%), cognitive behavioural therapies for OCD (18%), peer support (13%), cognitive behavioural therapies for tics (10%), and psychoeducation (9%).

Over half (55%) of parents stated they had a trusted source of information about TS and/or OCD. The most commonly mentioned sources were their health care provider, Tourette Canada, or the Tourette Association of America. Most (87%) felt that there was a lack of information resources for children with TS and/or OCD. Nearly all of the options for information that would be helpful were checked equally by respondents, with the highest number of respondents wanting help discussing their children’s educational needs with teachers and schools and strategies for school success, followed by behavioural treatment options. Parents requested information resources on how to cope with the diagnoses as a family.

Almost two-thirds (60%) of parents accessed resources to share with their child’s teacher. Information provided to teachers was most commonly the diagnosis, symptoms, and possible educational strategies. Almost two-thirds (62%) of parents stated that the recommended strategies were used in the school. Only 23% of parents accessed resources to teach other students in the classroom about TS and/or OCD as a way of addressing stigma or bullying. The most common resource used was an educational in-service in the classroom by the student or a volunteer from an advocacy organization.

When asked what they would like the Tourette OCD Alberta Network to focus their efforts on, all options were chosen nearly equivalently, but in descending order included: helping families connect with care providers that understand the condition, providing education about TS and/or OCD to teachers and in schools to improve school success, training more local health care providers to care for children with TS and/or OCD, providing education about TS and/or OCD to children and families, and connecting children and families with local peer and family support.

### Interviews

Interviews were conducted with ten parents of children diagnosed with TS and/or OCD. All ten interviewed parents were mothers. Four mothers resided in the Calgary zone, 2 in the Edmonton zone, 2 in the South zone, 1 in the Central zone and 1 in the North zone. Illustrative quotes were taken from these interviews and from the free text fields of the survey.

## Theme 1: Experiences receiving health care for TS and/or OCD in Alberta

### Accessibility

Accessibility to dedicated health care support was a key issue for the participants in this study requiring TS and OCD treatments for their children. Despite the small sample size, access issues were diverse and covered a range of difficulties.

#### The impact of cost and distance

Participants in this study reported that cost and affordability were determining factors for them to access health care professionals who were not covered by the government health care plan. At the outset of finding health care support, whether families had medical benefit packages was decisive in making healthcare decisions. The “exorbitant” costs of multiple visits to psychologists and therapists added to the problem of regularly attending vital sessions. Faced with the choice of terminating treatment for their child, parents just paid up: “So that’s $400 a week for a year. So I think there needs to be more in the province for families that can’t do that as well, that can’t afford to pay for it, I mean Dr. A got us through some pretty rough times, but we just paid.” (Parent Interview 1) Once families were able to directly receive treatment from government paid mental health professionals, the burden of cost lessened. The location of the TS and OCD clinic in Alberta is only convenient for families living in the center of the province. For these families, the impact of travelling to receive health care was minimal. Some made an 800-km round trip, taking more than 5 h each way, which was an intolerable distance and time investment. For others, it was a prohibitive cost, too.

#### First access: lack of a pathway

The participants often stated the first point of contact when trying to find and locate resources was their family physician. However, this did not always lead directly to a referral to a specialist. Family physicians were as likely to refer children to a specialist as they were not to refer at all. Some parents were assisted by child services, their child’s school, or they made the connection themselves. A coherent pathway to treatment was evidently absent, and on occasion parents met with the frustration of the inefficiencies of inter-health care practice communication. In such cases, access to treatment was gained by the persistence and diligence of individual health care workers. Moreover, the experience of initial engagement with health care services was, for some families, repeatedly reported as inadequate: “The nurse or my family physician, basically they sent the referral to Edmonton, they sent it back and said it’s no good; they tried it again, sent it back; they did a bunch of phone calls, were getting sent in circles, and then finally, actually phoned someone who happened to mention try Calgary and then that’s how she actually found the Movement Disorder Clinic, but it took probably 4 months of her phoning all over the place.” (Parent Interview 5) Though this was not the experience of all families, the lack of assistance navigating a route to treatment was viewed as atomized and “broken.” The struggle to find interconnected, continuous treatment underlined parents’ anxieties. The point of initial contact with a clinician was often the beginning of a difficult process to be directed to appropriate health care support: “Lack of navigation assistance or tools leave parents to wander through a segmented and broken system hoping to stumble into supports and therapies that will be effective.” (Free text from survey).

Participants felt hindered by the inertia of the health system: infrequent hospital appointments, misplaced referrals, lack of available resources, or a system designed for adult patients.

#### Timeliness

Notwithstanding these systemic issues with access, timeliness of treatment for some participants, once the health care system was accessed, was acceptable: “And the wait time didn’t matter, like to me it didn’t matter. We had waited so long, like what, even what was a year, like a year, okay, so we will be on the waitlist for a year, but it didn’t matter.” (Parent Interview 2) However, this belied the fact that participants were willing to overlook the unreasonably long wait times in the knowledge their child would eventually attend a specialized clinic. Wait times to see a psychiatrist ranged from 3 months to a few years. Tardiness of access to treatment was associated with the inefficiencies of the health care system, and even if families were detrimentally affected by this, their attitude to waiting indicated stoic acceptance: “Considering some people have to wait 3 years to see somebody, I don’t feel we waited too long.” (Parent Interview 6).

#### Working parents

Participants described the stress of full-time work as an obstacle to attending appointments. The inconvenience of day-time appointments, at clinics located hours from home, produced work-related pressures: “We gotta take off time from work, time from school because he [the health care provider] only comes in once a month. I can’t book it on my day off or his.” (Parent Interview 4) Participants who did not work outlined their relative advantage, one that also enabled them to do more independent research when finding resources that were not readily available in their community or from their family doctor: “When they went to school, I can sit down and start looking on the internet, and I could make appointments to my family doctor and go. I didn’t have to take time off work to do that…” (Parent Interview 1).

### Knowledge and skills

#### The knowledge gap

The findings from the interviews provide a picture of family physicians and healthcare workers who were at once obliging and disposed to helping families but simultaneously impeded by their lack of knowledge of TS and OCD—resulting in family frustration and, on occasion, confusion. This clinical paradox is most accurately underlined in the following participant’s comment: “My experience, overall, as far as their wanting to help and their attempts to try and find us solutions has been very positive but lack of expertise and lack of access to resources that they had has just made it a frustrating experience.” (Parent Interview 5).

Furthermore, our participants note that finding a pediatrician or a GP with the requisite knowledge was based, in some cases, on luck, resulting in an overriding sense of disappointment with the system. Misdiagnosis, lack of diagnosis, or an unwillingness to listen to families’ concerns about treatment choices were cited as other reasons for frustration by the participants: “Lack of properly trained therapists and doctor’s leads to long wait lists, misdiagnosis, and lost opportunities and patients go without interventions.” (Free text from survey) Most participants had fears regarding their own insufficient knowledge about medication, which were compounded by some doctors’ insistence on using medication rather than pursuing alternative treatments such as cognitive behavioral therapy: “They all want to just medicate your child.” (Parent Interview 8).

#### Level of expertise

Higher levels of satisfaction regarding quality of care were revealed in the participants' comments once they accessed clinicians with expertise in TS and OCD: “It took a little while to get help but once we were referred to [specialist], we've had good care since.” (Parent Interview 3) Participants’ experiences at the dedicated TS and OCD clinics were often rated as very good in terms of professionalism, seriousness, and access to dedicated resources: “When we finally got to the Movement Disorder Clinic because we finally felt like somebody actually took this seriously and didn’t just tell my child it’s all in your head.” (Parent Interview 5).

### Theme 2: Being a student with TS and/or OCD in Alberta

Our study inquired about the experiences of children and their families within the school system. Family experiences showed that schools often lack the levels of knowledge and preparedness to effectively support the learning of children with TS and OCD in the classroom. Moreover, our study found that participants repeatedly found themselves as agents of the schools’ path to understanding the health issues and comorbidities of these conditions: “Well for us, helping the school manage it was a big thing so I guess, I don’t know if there’s any kind of education that the health care system can provide to schools that have a child with Tourette syndrome….” (Parent Interview 6).

### Insufficient knowledge of TS and OCD

In different schools, strategies supporting children with TS and OCD either did not exist or were perfunctorily acknowledged, creating an environment in which children were unable to fully participate. An increasingly common narrative shared by participants was that principals, schools, and education boards were wilfully unsupportive due to the disruption attending to children with TS and OCD would cause: “Teachers would have a tendency to send my child out of the room or tell us to come get them because they’re being disruptive.” (Parent Interview 5) Conversely, some participants foregrounded principals and teachers who work within the limits of the system, employing educational strategies to enable children to achieve success, and were receptive to strategies to help in the classroom: “He can have a reader for his exams. He can take his exams outside the classroom. He has time, extra time if he is required, as needed, to help him be successful at school.” (Parent Interview 9).

### Parent as agent

Schools, however, operate in a context of promoting access to successful learning for all children in the classroom, and to ensure children with TS and OCD are included in achieving this objective, participants reported how frequently they shared educational strategies and their child’s diagnosis with the school. Parents’ interaction with the school and classroom happened in a variety of ways: parents gave in-class presentations, Tourette Canada’s handbooks were disseminated in schools, teachers were directed to specific online resources, mental health workers were brought into school to present to staff and students, parents had one-on-one chats with classroom teachers: “It’s really up to the parent to advocate and tell them what you know.” (Parent Interview 1) This parental advocacy role was a crucial vehicle for raising awareness of the needs of children with TS and OCD in the classroom. Participants who did not take on this intermediary role reflected that resources for parents took precedence over those for teachers: “no, resources for parents: number one,” (Parent Interview 10) Moreover, some felt teachers should already possess requisite knowledge, “Should be trained on how to deal with Tourette’s if they’re a special needs teacher.” (Parent Interview 8) These views describe participants who felt their or their child’s need was the priority, rather than the needs of teachers.

## Theme 3: The need for comprehensive information and support

### The difficulty of finding resources

Families receiving timely and relevant information was the key to enable participants to make decisions about the health care of their children. However, readily available information and resources, providing insight into their child’s condition, and offering a clear established pathway to access integrated healthcare services was rarely attainable. “I don’t think there’s enough information out there for people that is readily, you know, when their child is, I don’t even think they know where to go to get diagnosed.” (Parent Interview 1) There was an implied acceptance from some participants that they were responsible for gathering information and finding resources about their child’s condition, a situation which caused confusion and delay. Participants often located resources, but they were disparate, existing in different places, usually online or in books. A unified, single resource for TS and OCD was difficult to obtain. In contrast, when clear, comprehensive resource guides were accessible, the positive effect for parents was immediate, in that paying for a broad and full knowledge resource was acceptable “You’re going to have to pay for the online stuff or here’s the referral for psychologists who are dealing with it, here’s all this stuff for you.” (Parent Interview 2) The significance of being able to access specific types of resources and how they help families were repeatedly emphasized. The availability of an immediate online response to questions regarding tics was welcome, not least because the answer was non-judgmental. The private nature of independent learning about TS and OCD when relevant resources were provided was obvious: “I could put any of those [questions] out there and they would be answered with, like without being judged.” (Parent Interview 7) Furthermore, when the resource represented the insights of lived experience, it was invaluable.

### The foundation of help: peer support

The participants interviewed repeatedly revealed that coping with a diagnosis of TS and/or OCD was an isolating experience and that it causes uncertainty at a time when parents are increasingly feeling anxious: “Just trying to think, yeah, even for parents, there’s not a lot of peer support or educational information, other than what we can find online, which then you’re questioning on the validity of what you’re reading too.” (Parent interview 5) The need to find appropriate resources was also a quest, at times a desperate one, for support from other parents with lived experience. Underpinning parents’ search for guidance, vis-à-vis diagnosis, psychoeducation, or access to comprehensive health care was an appeal for assistance. Time and again, participants in our study bemoaned the absence of peer support. Peer support was formed ad hoc or occurred when a parent was already working in the education system and was able to form supportive groups who understood mental health issues: “Well I am really lucky. I was an educational assistant in the system when [my son] was diagnosed and so I have some really wonderful colleagues who have also become my friends and because they understand mental health issues because of their occupation, I was able to access a lot of support” (Parent Interview 10).

## Discussion

This study of people with lived experience with TS and OCD in Alberta has provided essential information for the planning and service provision of a province-wide support organization. It has highlighted the gaps in current services and needs of individuals with respect to healthcare, education, and support.

The Tourette OCD Alberta Network has pivoted to address the participant issues identified by families in the stakeholder consultation. Table 3 shows the direct response of the Tourette OCD Alberta Network to family need.

**Table 3:** How the Tourette OCD Alberta Network addressed needs identified by families with TS and/or OCD

Family need identified in interviews	How the TS Tourette OCD Alberta Network pivoted to need
Absence of coherent pathway to treatment	Albertans searching for assistance elicit immediate response from program coordinator via website
Inconvenience to working parents living afar	Consultations via telemedicine/videoconferencing
Clinicians’ skill gap	Webinars for healthcare professionals on psychoeducation and behavioural therapies for TS and OCD
Insufficient knowledge of TS and OCD in schools	Educational outreach program for teachers and classmates
Information about TS and OCD in unified, single resource	The Tourette OCD Alberta Network is attempting to be a central point of access to information related to TS and OCD and frequently co-occurring conditions through our website and newsletter. The website is a depository of information in a variety of forms and media. The organization of the website is user defined. Families and patients, health care professionals, and educators can access dedicated and differentiated resources by navigating to specific content. The program coordinator is available to field direct inquiries pertaining to, for example, clinical referrals and specific resources. In a drive to make information comprehensible, accessible, and timely, the Tourette OCD Alberta Network delivers regular online webinars. The webinars are publicized in our monthly newsletter and explicitly underline the events are for patients and families www.touretteocdalbertanetwork.ca
Peer Support	The Tourette OCD Alberta Network’s monthly newsletter regularly features articles, videos and profiles of real-world experiences of people living with TS and OCD. The strength that parents draw from other families’ experiences informs our intention to develop a peer support program

It is clear that one of the major services that the Tourette OCD Alberta Network should provide to meet the needs of our stakeholders is care navigation—helping people affected by these conditions find the medical, educational and informational resources they require. In order to successfully perform this role, the Tourette OCD Alberta Network and the services it provides must become widely known amongst patients, families and health care providers.

With only one center providing specialized care to a large geographic area, it is clear that flexibility of providers and patients with respect to service provision is essential. The increasing need to offer telemedicine appointments due to the global pandemic has forced changes in how services are provided in many healthcare systems. Within our own context, the pandemic facilitated the approval of the use of readily accessible video conferencing software for telemedicine appointments. The provision of videoconferencing technology enables the expertise that is focused in Calgary to be accessible to the wider province of Alberta, thereby extending specialized care to many more families. Partnerships with local clinics, to perform physical monitoring of blood pressure, heart rate, height, weight, waist circumference and the extrapyramidal system, are essential for the safe provision of care.

Videoconferencing has been shown to be an effective way of providing behavioural therapies for both TS and OCD. In a randomized controlled trial comparing CBT for OCD delivered by video-conferencing to self-help or having to wait for treatment, telemedicine resulted in a significant reduction of OCD symptoms [5]. A study of twenty children randomly assigned to receive CBIT face-to-face or delivered by videoconferencing found that both treatment delivery modalities resulted in significant tic reduction with no between group differences [6]. Tic reduction was comparable to CBIT randomized control trials, in that the mean YGTSS score reduced by 33% from baseline. The study also reported children and parents had strong working relationships with their therapists.

Families participating in our stakeholder consultation discussed the difficulties they had finding experienced care providers locally, and the out-of-pocket expenses they incurred seeking private services. In an effort to try to improve access to knowledgeable care providers, we have developed and recently launched a continuing professional development webinar series on behavioural treatment of TS and OCD for mental health therapists working within our government-paid system of mental health clinics province-wide. Initial uptake of this program has been excellent, and we hope that this skill development opportunity will translate into greater access to appropriate care.

Both the survey and interviews highlighted the need for advocacy in schools for students affected by TS and OCD. This includes education with teachers and classmates to promote understanding and reduce stigma and developing strategies to promote educational success. Data on educational attainment suggest that strategies to promote school success are needed for people living with TS. A population-based Canadian study found that fewer people with TS had post-secondary education compared to the general population [7]. In addition, fewer adults with TS were employed compared to the general population. The impact of OCD on educational achievement in relation to secondary school and university is pronounced [8]. In a nationwide register-based Swedish study assessing educational attainment after compulsory school, compared with population controls, individuals with OCD were significantly less likely to achieve the assessed educational levels during the 22-year study period. In the adjusted models, individuals with OCD were 57% less likely to complete upper secondary school, 28% less likely to start a university degree, 41% less likely to finish a university degree, and 48% less likely to complete postgraduate education compared with those without OCD.

Individuals with tics may be considered less socially acceptable by both peers and teachers. A review of studies designed to understand the impact of educational information about TS found that giving information about TS to peers resulted in more positive attitudes toward a person with TS, regardless of age, gender or severity of tics [9]. One study found that a 2-h workshop to teachers about TS resulted in significant increases in knowledge [10]. In an evaluation of a classroom presentation about TS, it was reported that after the presentation, classmates increased their levels of empathy for the individual with TS and expressed concern for the individual’s well-being in the classroom [11]. Classmates’ attitudes to TS is influenced by the perception of the majority view of TS in the class. Therefore, empathy in the classroom rises when the majority view is more accepting of TS, which was appreciable after the presentation evaluated in the study.

The findings of this study and the proposed solutions are of relevance to health care providers or organizations providing services to children with other mental health disorders. While the participants in our study were Albertans affected by TS and/or OCD, many of the health care needs and proposed solutions highlighted in this study are applicable across locations, disorders and health care systems. Strategies to support educational needs and peer support seem most relevant. For example, ADHD is the most common mental health disorder among children worldwide. A population-based study in Scotland found that children with ADHD have more unexcused absences, are more likely to be excluded from school, have worse educational outcomes, leave school earlier, and are at higher risk of special educational needs [12]. A systematic review of qualitative evidence of parenting experiences of living with a child with ADHD highlighted many of the same findings of our study, including the difficulties of navigating the system and the need to provide appropriate supports [13]. Care navigation, educational outreach and peer support would likely be of great benefit for families affected by ADHD.

A qualitative study on parental perceptions and experiences with children with TS in Australia found that their greatest needs were for immediate, accessible, and comprehensive information on TS for parents, as well as education of those within the school system, including teachers, principals, classroom aids, school administrators and educational policy makers [14]. Parents described the social isolation they experienced as a result of their child’s TS diagnosis and the importance of having a bridge to the outside world to counter the isolation, distress and difficulties they faced associated with the lack of understanding of TS by others. Social support organizations for TS, mother support groups, and understanding medical professionals were of key importance. The commonality of findings between our stakeholder consultation and this study in another healthcare system on a different continent suggest that our findings are relevant to others affected by TS and OCD outside of Alberta.

Limitations of the current study include the recruitment of parents who were receiving services at the Alberta Children’s Hospital TS and OCD clinics for participation in the qualitative interview portion of the study. As these parents were receiving specialist services, their views may not reflect those of parents and families who have not been able to receive specialist care, or who have less severe symptoms.

## Conclusion

This study identifies the diversity of factors determining how families requiring TS and OCD treatments for their children in Alberta access the health care system, navigate challenges in the school system and seek out health information and support. TS and OCD require comprehensive and specialized care, and It is evident that families’ experiences of navigating a pathway to reach this care are uneven. In providing support for people affected by these conditions, the Tourette OCD Alberta Network seeks to recognize the different types of help families need. The on-going aim is to ensure this community of families is not only aware of the services the Tourette OCD Alberta Network provides but is also informed as to how these services can improve and positively impact their lives. Future work will include assessment of the impact of the services the Tourette OCD Alberta Network provides on health outcomes.

## Supplementary Information

**Additional file 1.** Survey for people with lived experience with Tourette syndrome or OCD. Survey questions.

## Data Availability

The datasets used and/or analysed during the current study are available from the corresponding author on reasonable request.
