# Supplementary material for: Developing a provincial patient support network for children and families affected by Tourette syndrome and/or obsessive–compulsive disorder: results of a stakeholder consultation
Source: Child Adolesc Psychiatry Ment Health. 2021 Jun 16;15:29. doi: 10.1186/s13034-021-00383-5 (PMC8208059; doi:10.1186/s13034-021-00383-5)
Supplement: Supplementary file 1 — Additional file 1. Survey for people with lived experience with Tourette syndrome or OCD. Survey questions. [file 13034_2021_383_MOESM1_ESM.pdf]

Survey for People with Lived Experience with Tourette Syndrome or OCD

Study Title: ***The Tourette OCD Alberta Network: Stakeholder consultation***

Year of Birth: ________________

First three letters/digits of postal code: ________________________

**Interview questions (estimated 15 minutes)**

A. Access & navigating mental health care for your child

Questions

1. Which of the following types of physicians have you seen to receive care for your child's diagnosis of Tourette Syndrome and/or Obsessive Compulsive Disorder? Check all that apply.

☐ Family physician
☐ Pediatrician
☐ Psychiatrist
☐ Neurologist
☐ Other, please specify ____

2. What type of physician is currently providing on-going care for your child for their diagnosis? By on-going care, we mean writing prescriptions and providing regular follow-up visits.

☐ Family physician
☐ Pediatrician
☐ Psychiatrist
☐ Neurologist
☐ Other, please specify ____

3. What is the distance in kilometers you have to travel to obtain on-going medical care for your child?

____

4. Have you seen any (non-physician) healthcare professionals for your child's diagnosis of Tourette Syndrome and/or Obsessive-Compulsive Disorder?

☐ Yes ☐ No

5. What types of other health care providers did you see? Check all that apply.

☐ Psychologist

☐ Social worker

☐ Occupational therapist

☐ Physical therapist

☐ Mental health counsellor

☐ Family therapist

☐ Other health care provider

☐ None of the above

6. How did you find out about these health care providers?

☐ Referred by a physician

☐ Notified of provider by patient support organization

☐ Recommended a friend

☐ Access Mental Health

☐ Internet search

7. Did you experience any difficulties finding a person with the right skills to see your child?

☐ Yes ☐ No

8. Did you have to pay a fee to see this person? If you are seeing more than one non-physician health care provider, please answer the question for the provider you see most often.

☐ Yes ☐ No

9. How long did you wait to be seen in months?

____

10. How far do you have to travel in kilometers to see this person?

---

11. What types of services are provided? Check all that apply

☐ Cognitive behavioural therapies for tics, for example, Comprehensive behavioural intervention for tics, habit reversal therapy or exposure and response prevention

☐ Cognitive behavioural therapy for OCD, for example, exposure and response prevention, or mindfulness-based cognitive therapy

☐ Family therapy

☐ Psychoeducation

☐ Peer support

☐ Parent training on how to manage problems with anger or aggressive behavior

☐ Other, please specify ____

B. Searching for information about Tourette and OCD.

Questions

1. Do you have a trusted source of information about Tourette syndrome or OCD?

☐ Yes ☐ No

If yes;

1a. What is it?

1b. Where did you hear about it?

☐ Internet search
☐ Recommended by physician or other health care provider
☐ Recommended by a patient support organization
☐ Recommended by a friend

2. Do you feel there are any information resources that are currently lacking for children and families affected by Tourette Syndrome and OCD?

☐ Yes ☐ No

If yes;

2a. What types of information would be helpful? Check all that apply

☐ Information on the condition and what to expect over time

☐ Medical treatment options

☐ Behavioural treatment options

☐ Places children and families can receive care for their diagnosis of Tourette Syndrome and/or Obsessive Compulsive Disorder

☐ Strategies for school success

C. Resources for success in the classroom

Questions

1. Did you access any resources to share with your child's teacher about their diagnosis, symptoms, or educational strategies?

☐ Yes ☐ No

If yes;

1a. What were they?

1b. Were they helpful?

☐ Yes ☐ No

1c. Was the teacher receptive to receiving this information?

☐ Yes ☐ No

1d. Were the recommended strategies used in the classroom?

☐ Yes ☐ No

2. Did you access any resources to teach other students in the classroom about Tourette syndrome or Obsessive-Compulsive Disorder as a way of addressing stigma or bullying?

☐ Yes ☐ No

If yes;

2a. What were they?

2b. Were they helpful?

☐ Yes ☐ No

D. The Tourette OCD Alberta Network

Questions

1. The goal of the Tourette OCD Alberta Network is the increase the capacity to care for children with Tourette syndrome and OCD in Alberta within the communities they live in, and to connect patients with the resources they need to manage the symptoms of these disorders. What do you think is the most meaningful way the Tourette OCD Alberta Network can help your family?

____

1a. What would you like the network to focus on? Check all that apply

☐ Providing education about Tourette syndrome and OCD to children and families

☐ Helping children and families connect with care providers that understand the condition

☐ Connecting children and families with local peer support

☐ Training more local health care providers to provide care for children with Tourette syndrome and OCD

☐ Providing education about Tourette syndrome and OCD to teachers and in classrooms to improve school success

☐ Other, specify ____
